# Molecular Biology of the Cell

**Published:** 1983-10

**Authors:** J. D. Davies

**Affiliations:** Histopathologist University of Bristol


### Book Review

### MOLECULAR BIOLOGY OF THE CELL

**B. Alberts, D. Bray, J. Lewis, M. Raff, K. Roberts and J. D. Watson**

**Garland Publishing Inc., New York and London. 1983 1146 pp., xxxix, +35, in soft covers Price £12.50**

This is not yet another routine book review. No free copy has passed from your Editor to an overburdened hack, who, if flattered, has then to compose a politely phrased summary. Your correspondent voluntarily purchased this 1200-page offering without coercion. OK, you may say, so what?

For generations in medicine we have all been hammered into submission to believe in Virchow's cellular viewpoint. That is all very well. In any case academics will swallow almost anything if it's their job. Even if the botanists of the 18th century, the well-intentioned of the 19th century and the latter-day enthusiasts of this century believed in the cell, is it an approach that is truly relevant? Such must surely be the attitude of today's sceptical medical students, and of many of their now middle-aged predecessors.

Well, let a battle-scarred predecessor speak. Even a conventional diet of comparative and classical morphology, combined with exposure to pre-fashionable Eltonian ecology, and a native distrust of big-business 'nouvelle vague' biology has not blinkered your reviewer to the virtues of Alberts' *Molecular Biology of the Cell*.

This is undoubtedly a landmark for the student and his teacher in the field of preclinical medicine. Many American undergraduate texts in biochemistry and molecular genetics clearly outsell competing English publications. Only English textbooks in immunology remain popular, and effective sellers in this field. The successful texts in these areas eschew traditional academic contrapuntal argument. The new best-selling formula involves clarity of presentation, the effective use of multi-coloured diagrams and the detailed exposition of basic processes. Such didactic approaches suit undergraduates today, and certainly ease the hardship for their seniors needing a refresher course.

All these virtues are combined in Alberts' *Molecular Biology of the Cell*. This is *the* book which manages to integrate the essentials of modern biochemistry, immunology, embryology and the basis of anatomical structure. Furthermore, it incorporates the subtle derangements which are the foundation of general and special pathology, and hence of clinical medicine. For the vaguely uneasy practitioner who does not always understand his students, and feels in need of a brush-up, this book promises good reading. Take courage, here is a clear exposition of modern cell biology. The behaviour of cells, intercellular matrix, their inter-relationships, messages, genetics and significance for medicine are all considered. As for cost, even as bed-time reading, it is ridiculously cheap. Do try it.

J. D. DAVIES
Histopathologist
University of Bristol

